# ‘I have more control over my life’: A qualitative exploration of challenges, opportunities, and support needs among autistic university students

**DOI:** 10.1177/23969415211010419

**Published:** 2021-05-17

**Authors:** Matthew Scott, Felicity Sedgewick

**Affiliations:** School of Education, 1980 University of Bristol, UK; School of Psychology, University of Cardiff, UK; School of Education, 1980 University of Bristol, UK

**Keywords:** Autism, higher education, mental health, support

## Abstract

**Background:**

Autistic people are known to experience more mental health issues than non-autistic people, and the same is true among university students. These difficulties can have long-term consequences, such as dropping out of university and unemployment. Understanding the challenges autistic students face can help institutions to better support this group, while allowing celebration of the opportunities higher education offers.

**Methods:**

12 autistic university students took part in semi-structured interviews about their mental health, the impact of university on their mental health, and their experiences of support while in higher education. Interviews were subject to thematic analysis.

**Results:**

Three key themes were identified from autistic student accounts: Relationships, Independence, and Support. While each of these encompassed positive and negative elements, Relationships were described as tying everything together – when these were supportive, things went well, but when they were characterized by stigmatizing attitudes, students experienced much greater difficulties at university.

**Conclusions:**

Autistic students can and do thrive at university, as shown by many of our participants. However, all faced significant challenges with their mental health at times, and experienced varying levels of support. Improving autism knowledge among staff, with emphasis on enabling better relationships, would make a significant difference to the autistic student experience.

## Introduction

Autistic people (terminology used in line with community preference, Kenny et al., 2016) are more likely to experience challenges with their mental health than the non-autistic population – a pattern evident across the lifespan from childhood and adolescence (White et al., 2009), to adulthood and old age (Hand et al., 2020). The most common mental health conditions among autistic people are anxiety and depression, with up to 80% meeting the clinical criteria for anxiety at some point in their lifetime (Lever & Geurts, 2016) and around 40% meeting criteria for depression at any given point (Hollocks et al., 2019). These issues are more pronounced in individuals without cognitive impairment, possibly because they are more aware of the difficulties they’re facing (Sterling et al., 2008). Alongside anxiety and depression, autistic people are more likely to develop eating disorders (ED) than their non-autistic peers (Sedgewick et al., 2020), and to experience post-traumatic stress disorder (PTSD), especially complex-PTSD (Haruvi-Lamdan et al., 2018; Rumball et al., 2020). While there are some measurement issues around assessing autistic traits in clinical populations due to overlapping diagnostic criteria and symptom profiles (Huke et al., 2013; Westwood & Tchanturia, 2017), evidence for the comparatively high frequency of these diagnoses among autistic people is robust. Crucially, these poor mental health outcomes are common even among those with ‘good’ employment and relationship outcomes in adulthood (Gotham et al., 2015), leading to high rates of suicidality among autistic people, especially those who are considered cognitively able (Cassidy et al., 2018).

Research has shown that autistic people are likely to struggle to access professional mental health support for a variety of reasons: limited understanding of autism-specific presentations and insufficient tailored mental health treatments, alongside overly complex systems which are hard to navigate (Crane et al., 2019; McMorris et al., 2019). Furthermore, there is growing evidence that even when autistic people do access care, they respond differently than non-autistic people to common psychotherapeutic interventions (Kinnaird et al., 2017; Tchanturia et al., 2016), which impacts the efficacy of these mental health treatments.

University is known to be a psychologically challenging time for all young people – it is a time of transition, increasing independence and academic demands, along with the social challenges of forming new relationships (Briggs et al., 2012; Clark, 2005). Mental health difficulties are common among students in higher education, especially anxiety (Bayram & Bilgel, 2008; Storrie et al., 2010) and depression (Ibrahim et al., 2013). These challenges are often intensified for autistic young people, for whom research indicates the transition to adulthood is particularly difficult (Cheak-Zamora et al., 2015; Lambe et al., 2019). Equally, the social aspect of university - meeting new people, trying to make friends and navigating new living arrangements - has been specified as intimidating and difficult for many autistic students (Lei et al., 2020). Challenges with social relationships are a diagnostic criterion for autism (American Psychiatric Association, 2013) and have been theorized as a key underlying mechanism for the condition, as in the Social Motivation Theory (Chevallier et al., 2012). This theory argues that autistic people have lower levels of social motivation than non-autistic people, creating a cycle of reduced engagement and negative social feedback, leading to social anxiety and other mental health issues. It is worth noting that aspects of this theory have been challenged – autistic girls have been found to have higher levels of social motivation than boys, for example (Dean et al., 2014; Sedgewick et al., 2019). Furthermore, recent research into autistic-autistic communication has highlighted that these are just as successful as those between non-autistic people, suggesting that difficulties may arise in cross-neurotype interactions rather than being an inherent feature of autistic people (Crompton et al., 2020).

Regardless of theoretical critiques, most autistic young people report social difficulties at school, including being bullied, ostracised, or ignored by peers (Carpenter et al., 2019; Hebron & Humphrey, 2014; Rowley et al., 2012). Being victimized at school is known to be associated with worse mental health, especially anxiety and depression (Landstedt & Persson, 2014; Turner et al., 2013). This relationship between poor social experiences and mental health is also true for autistic young people (Ashburner et al., 2019; Rodriguez et al., 2021). It therefore makes sense that social relationships would be a crucial area of concern for autistic students at university.

There is a growing body of evidence that autistic university students do indeed struggle with several aspects of their time in higher education. New situations inherent in university life, relationships, and demands on information processing and time management can present challenges for some autistic people (Van Hees et al., 2015). These issues, combined with feeling different to their peers due to being autistic, and often facing a lack of understanding of their needs (Lipson et al., 2020), can contribute to negative long-term outcomes and mental health (Cage et al., 2018). Indeed, despite high levels of academic confidence, many autistic students report feeling isolated, stressed, anxious and depressed (Gurbuz et al., 2019; Volkmar et al., 2017). This can lead to students feeling like leaving their course is the only option (Cage & Howes, 2020), as seen in high drop-out rates among autistic students and frequent difficulties entering and maintaining employment (Ohl et al., 2017; Vincent, 2020).

The few papers which have examined the specific academic, organizational and mental health-related support needs of autistic students at university have generated a wide range of suggestions (Accardo et al., 2019). Recent reviews of university-provided formal support strategies, however, found their use and effectiveness to be highly idiosyncratic, meaning they need to be personalized (Anderson et al., 2019; Mulder & Cashin, 2014). Further, these options are only available to those who are comfortable disclosing their autistic status, accessing, and using support, which many are not (Anderson et al., 2018). Contrastingly, some students describe deliberately making themselves hyper-visible as autism advocates to raise awareness in their academic community (MacLeod et al., 2018), but such autistic voices regarding experiences of university support remain strikingly absent - something this paper seeks to address.

In relation to informal university-based support, those who establish and maintain friendships and romantic relationships report better subjective wellbeing than students who do not have this support network (Bailey et al., 2020; Lamport & Turner, 2014). Also, despite evidence that autism-stigma exists in universities and has a negative impact on autistic students, research suggests that if peers are made aware of the diagnosis, they rate an autistic peer more positively and this increases acceptance (Matthews et al., 2015; Nevill & White, 2011). Encouragingly, knowledge of autism appears to be increasing among students and staff, with correspondingly lower levels of stigma (Stronach et al., 2019; White et al., 2019).

While there is a growing evidence base documenting the mental health and other challenges autistic students experience at university, we currently know little about their engagement with and views on support for these issues. Understanding these experiences may not only improve student retention, but highlight their needs to faculty, staff, and Wellbeing/Support Services, creating a better experience for autistic students. The present study aimed to explore these questions through qualitative interviews with students at a variety of stages of study. The main research question was ‘What are the mental health experiences of autistic students at university, and how does university contribute to or mitigate these experiences?’

## Methods

### Participants

Twelve autistic students from the same British university participated in semi-structured interviews. Eight participants were female, three male, and one person identified as non-binary (Mean age = 24.08 years, *SD =* 5.23, range: 19–36). Eleven participants had a formal autism diagnosis, from a range of clinical services, and one had a working diagnosis from their general practitioner. Nine participants had at least one additional mental health disorder, with anxiety (*n* = 8) and depression (*n* = 7) the most common. Six participants had more than one mental health disorder (see Table 1 for this and other demographics).

**Table 1.** Participant demographics.

Participant pseudonym	Age	Gender	Ethnicity	Subject (degree type)	Year of study	Diagnosed mental health conditions
Jennifer	33	Female	Latina	Education (PhD)	2	None
Angela	20	Female	White British	Childhood Studies (UG)	2	Anxiety, depression, OCD, ED
Chloe	21	Female	White British	Anthropology (UG)	2	None
Edward	24	Male	White British	Music (PGCE)	1	Depression
Theo	20	Male	White European	Chemical Physics (UG)	1	None
Teresa	24	Female	White British	Psychology of Education (PG)	1	Generalised anxiety disorder
Emily	19	Female	White British	Mathematics (UG)	1	Anxiety, depression
Michael	23	Non-binary	White British	Veterinary Science (UG)	2	Anxiety, depression
Patricia	24	Female	White British	Music (PGCE)	1	Anxiety, depression, OCD, ED
Aran	24	Male	White British	Mathematics (PhD)	3	Generalised anxiety disorder
Louise	36	Female	White British	Social Work (PG)	2	Anxiety, depression, ED, ADHD
Janet	21	Female	White British	German and Spanish (UG)	3	Anxiety, depression

Recruitment took place through advertising the study to participants from a larger quantitative study cohort within the same funding grant (data currently unpublished), with recruitment running from early September to late October 2019. This was advertised through departmental mailing lists, the Disability and Wellbeing Services, and flyers placed in departments across the university. Ethical approval was granted by the School of Education’s Ethics Committee, and all participants provided informed consent before taking part. Initially 28 participants expressed interest in that study, but by the time of invite to interview (February 2020), six had dropped out of the cohort following non-response to one or two rounds of data collection. Participants were invited up to three times, and if they did not respond then they were not pursued further.

It is worth noting that our sample was majority non-male – inconsistent with the general diagnosis patterns within autism which has a 3:1 male:female ratio (Loomes et al., 2017). This means that our sample is not representative of the majority of those diagnosed as autistic, although there are recognized gender biases in the diagnostic criteria (Gould, 2017). However, this piece of exploratory qualitative work does not seek to present the experiences of all autistic people. Further, with more women than men attending higher education (*Who’s studying in HE? | HESA*, 2019), and taking part in university and mental health research (Woodall et al., 2010), this balance is not surprising.

### Materials

*Demographics* data was already held as part of the larger study participants were enrolled in, and covered aspects such as age, gender, autism status, educational status, and physical and mental health.

The *semi-structured interview* questions covered the positive and negative impacts of university life on participants mental health; changes in this over time; mental health support at university; and recommendations for improving support. The interview schedule was co-produced with two autistic consultants, who collaborated on initial ideas for questions that would have been relevant to them during their time in higher education, and then reviewed a draft interview schedule for clarity and accessibility of language. Following these discussions, the interview included questions such as: “Did anything change about your mental health when you came to university?”, “What aspects of university did you find had negative/positive impacts on your mental health?”, and “Have you asked anyone for help with your mental health while you’ve been at university?”. All main questions had planned prompt questions, with specific suggestions from the autistic consultants to make things as clear as possible for participants, such as: “Have you had any issues with anxiety or depression for example?” and “Are certain periods more difficult than others for you?”.

### Procedure

Participants were given the opportunity to be interviewed face-to-face, via online video chat, via online chat with audio only, or via live chat (typed) on the recommendations of the autistic consultants. This meant that participants were able to engage with the research in ways which were most comfortable and conducive to their involvement. Planning for, and accommodating, the communication preferences of autistic people in research can have a positive impact on the authenticity of the data collected, as it allows participants to communicate more effectively (Howard & Sedgewick, in press). Due to the outbreak of COVID-19, only two were interviewed face-to-face before health risks were considered too great. Interviews took place between February and June 2020, lasting on average 28 minutes (range: 19–43 minutes). Interviews were audio recorded and transcribed verbatim, before being checked for accuracy by the research team. Three participants sent emails after interview with clarifications and/or further details, which were subsequently added to their interview transcripts. Participants each received a £20 Amazon voucher.

### Data analysis

Transcripts were subject to inductive thematic analysis i.e. without any theoretically grounded, predetermined themes. This followed the six steps outlined by Braun and Clarke (2006), namely (1) data familiarisation, (2) generation of initial codes from semantic content, (3) searching for themes which acted as descriptive overviews of codes, (4) reviewing themes through discussion between the authors and the autistic consultants, (5) defining and naming themes, again as a collaborative process, and (6) report production. The analytic process was iterative in nature, with the first author coding transcripts line-by-line after initial data familiarisation, and the other team members independently coding between 20–40% of transcripts, blind to first author coding to counter potential analytic biases. The team then met to reach consensus on the themes and subthemes via discussion.

The authors wish to highlight that they approached this process as “informed outsiders”, as non-autistic researchers who nevertheless are highly involved and invested in the autism community and the wellbeing of autistic people. Our autistic consultants felt that our analysis was an appropriate interpretation and representation of the interviews.

## Results

**Figure 1.**
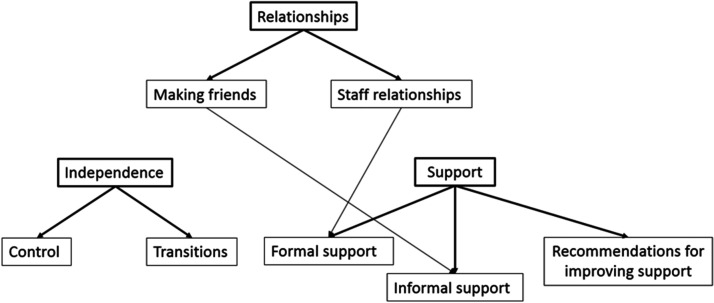
Thematic map of themes and subthemes. Bold lines denote direct subthemes, thin lines denote other connections between subthemes.

Three overarching, interacting themes were identified – *Relationships, Independence,* and *Support*. Themes and sub-themes within these areas are outlined in Figure 1. Quotes are italicized, with a participant pseudonym and year of study in brackets.

### Relationships

The *Relationships* theme was split into two subthemes: *making friends* and *staff relationships*. Participant’s relationships were repeatedly mentioned as crucial to how well – or poorly – things were going. Most participants experienced positive relationships at university, which centered firstly on making understanding friends, and secondly on finding staff with knowledge of autism and a proactive approach to support. These positive relationships created opportunities for autistic individuals to be open about their mental health and seek support when required. However, challenges arose in initially forming these bonds for a range of reasons.

*Making friends.* Many participants found forming relationships difficult before university – due in part to pressure to socialise and having to mask their autistic traits: “I think where the autism was so hidden, I’ve always been very good at *being*** **sociable and *being* outgoing … [from that] my mental health has really suffered.” (Patricia, Y1).

Despite earlier problems making friends, several participants were excited to talk about how much more genuine their university friendships were – “I’ve got a group of friends now that I’m solid with and they understand me and we get on well” (Janet, Y3). For many, this was because university was an opportunity to be themselves “I can just fully be myself and they will accept that” (Angela), which had profound positive impacts on their mental health: “My second year of uni I was probably the most well I’ve been for 10 years, ‘cause I lived with a group of really really supportive people” (Patricia, Y1).

Considering the value placed on friendships by participants, it was unsurprising that many also talked about *how* they met their friends. Experiences varied, with some students finding socializing easier than others, often for reasons linked to being autistic. Several participants discussed how aspects of university structure – namely course organisation and accommodation – played a major role in their ability to make and maintain friendships. Those with more practical and group-based elements talked about forming relationships with their course mates due to “the nature of the course” (Louise, Y2). Others had more difficulty however, on account of more independent study patterns which did not provide opportunities for structured social interaction, which they felt they needed as “going up to someone to just talk is really hard, I’m too nervous” (Aran, Y3). Within halls, while one participant was “really lucky” (Chloe, Y2) with their relationships, many found the social expectations and sensory overload therein unmanageable: “If you’re not a social butterfly, and you don’t go out drinking every day, if you don’t go out and if you’re sensitive to noise and smell then halls is probably the worst place you can go for mental health.” (Edward, Y1).

Joining societies offered an alternative opportunity to meet like-minded people with similar interests. This was appreciated by some as it gave a focus to their time together, allowing them to foster and manage successful relationships: “That’s pretty much where I’ve made all my friends, is through cheer … That’s really helped me a lot with my confidence.” (Angela, Y2).

However, access to societies was not straightforward for all participants, with reported difficulties signing up for societies (“I’d find it nice it you could go to one of these societies with a buddy … I don’t really know how to join them” (Theo, Y1)), and executive functioning challenges balancing the demands of clubs and their course – “There’s so much to keep track of, work and societies and friends…it can be overwhelming” (Janet, Y3).

While all participants had positive peer relationships, particularly in contrast to being bullied as some had in high school, many talked about experienced barriers to socializing, and how these led to them feel socially isolated. The start of university appeared key in forming participant’s impressions for the future: “I actually wanted to have some friends … the [fresher’s] fair shocked me … so then I didn’t really know how to interact with other students” (Theo, Y1).

Some also found that expectations of ‘being a university student’ - including pressure to go clubbing or other activities which triggered their sensory sensitivities - made them feel isolated, and like they needed to use alcohol as a social ‘crutch’:

I just felt like I couldn’t enjoy a lot of social situations in first year without being drunk, ‘cause they were so fucking loud, or stressful. That was something that made me quite depressed: I’d come away from hanging out with new people or going on a night out or something being like “oh my god I’m never gonna like fit in”. (Janet, Y3)

*Staff relationships.* Participants who built relationships with autism-knowledgeable staff – particularly Personal Tutors – generally reported feeling more comfortable approaching them for support, and that the support they received was better. One participant noted that “it helps to have that expertise” (Aran, Y3), and another revealed relief at having a tutor with previous experience in the field: “They said “we can give you a tutor who knows autism” and I was like “great, go for it”” (Teresa, Y1).

While this awareness was important, flexibility and proactivity in approach also held value for many participants, as they felt they were treated as an individual rather than as a stereotype: “I don’t really know if its adapted because of autism or just because they do it person-centred anyway … they kind of saw that I liked certain things and didn’t like other things.” (Angela, Y2).

Sadly, a lack of autism knowledge was often described as the defining feature of student-staff relationships – an issue of heightened pertinence when involving Personal Tutors or other staff positioned to take up a direct role in student’s mental health support. I said “just to let you know, I’ve just been diagnosed with autism”… he was like “I don’t really agree with the premise of diagnosing autism, I think that it creates a self-fulfilling prophecy and it will just make you act more autistic”. (Angela, Y2).

This type of undermining response from some staff perpetuated fears of being stigmatized for being autistic by all staff – “I don’t really want my lecturers knowing ‘cause they sort of, start to talk to you differently” (Chloe, Y2) *–* and subsequently several participants said that they were reluctant to seek support, even when they knew it was available. In a similarly worrying vein, one participant recounted how a lecture about the difficulties of being friends with an autistic person “made me feel quite isolated thinking about all the reasons it could be difficult to be friends with “someone like me”” (Angela, Y2). In this case, a lack of autism awareness from staff actively contributed to anxieties around peer relationships, as well as making the student doubt how approachable her lecturer would be if she needed support.

### Independence

The Independence theme was split into two subthemes: *control* and *transitions*. Overall, participants discussed an array of enabling and challenging facets to changes in their level of independence at university. Most viewed the opportunity to become increasingly self-sufficient as a gratifying shift in lifestyle – especially when concurrent with the improved peer relationships many were experiencing. They thus felt supported to engage with university life in ways that they had not always envisaged, which had a positive impact on their mental health: “I found a really good balance between doing extra-curricular stuff that I love, but not doing too much so that I could also focus on work … found that sweet-spot really” (Patricia, Y1).

*Control.* One factor that contributed to this was having more control over their life, and being able to follow self-directed routines: “it really suits me ‘cause I have control of exactly, when I can do what, like I do really like that flexibility you get” (Teresa, Y1). Furthermore, the associated “freedom” to choose who you spend time with – “if you’re worried … you just don’t have to be round those people” (Janet, Y3) *–* was preferable for participants when compared to school, where the members of your social network are relatively fixed and consequently difficult interactions common. Beyond the benefits for mental health, one participant noted the impact ‘taking back control’ had on their relationships at university and beyond: “I’ve generally found that my relationships with people … have got a lot better because I have kind of that space … I’ve changed and become more confident as well” (Michael, Y2).

*Transitions.* Alongside the expected day-to-day challenges of living independently at university, participants highlighted that periods of change and transition were especially difficult for them. Even if they were generally confident in university life, transitions to and from university often exacerbated problems – “it was more like *changing* to uni that was hard, rather than actually being at uni” (Chloe, Y2). This difficulty centered on the establishment of a routine for most participants (“I didn’t have a schedule and I couldn’t really have a nice routine” (Edward, Y1)), despite these routines being a significant positive once settled into. Some noted the adverse impact such a change in lifestyle had on their mental health – such as responding to uncertainty with aggression, “I felt a lot more aggressive inside… it might just be a new lifestyle I guess” (Theo, Y1) or increased anxiety*:* “it’s a lot of change and that’s difficult to manage, I worry a lot” (Chloe, Y2).

Participants also found that managing mental health became increasingly burdensome at transition times due to overlapping demands of home and university life: “there’s so many more demands on me, expectations … I’m kind of more in control of that at university” (Teresa, Y1). Similarly, time at home could intensify mental health problems when returning to university due to a ‘buildup’ of issues: “My self-harm is generally worse when I come back to uni ‘cause I can’t really self-harm at home” (Angela, Y2).

Many participants talked about trying to prepare for these transitions, which often involved the friends they had made at university: “my friends help me stay fed, stop my ED getting worse” (Emily, Y1). Such contingencies made transitions feel more predictable, ameliorating some of the mental health challenges they were experiencing, while providing satisfaction that they were able to put these in place for themselves. Similarly, those that had more preparation time before starting university reported more positive transitions to the new lifestyle: “I wasn’t ready to go to university when I was 18 … I’d had an extra year to plan and prepare stuff, so, whilst it was strange and kind of difficult, it was OK” (Emily, Y1).

### Support

The final overarching theme, *support,* was split into two subthemes – *formal and informal support,* and *recommendations* for improving support.

*Formal support*. Participants spoke varyingly about the formal university structures, systems they had accessed to support their mental health. A range of challenges to improving and maintaining positive mental health were discussed, such as tackling high-pressure assessment (“presentations are an extra stress” (Emily, Y1)), which were often adapted to suit student’s needs: “I don’t have to do exams anymore, which is like a massive thing ‘cause that was so much anxiety …” (Angela, Y2).

One of the key challenges to accessing formal mental health support our participants encountered related to organization of both the transfer to new services, and between services within the university itself. They often had to initiate and manage this themselves. One participant, for example, was told she did not qualify for support in the new area because she “had not tried to kill [herself] recently” (Angela, Y2) and so was not considered an extreme case. Accessing the right *university* support was also not straightforward, with participants “going back and forth trying to get hold of the service I need” (Emily, Y1) and teams not being connected: “Disability services is completely separate to wellbeing, and that’s like a bit of a minefield, honestly” (Aran, Y3).

Participants noted that issues finding autism-tailored support on the NHS – “it just doesn’t exist [on the NHS]” (Teresa, Y1) *–* extended to university. Without services recognizing the “extra layer” (Teresa, Y1) autism represents, many felt that their mental health support had been ineffective because staff would “just assume” that something is appropriate for an autistic person without asking them.

Conversely, positive experiences of support occurred when – instead of making such assumptions – university services approached participant’s distinctive cases holistically. For one participant, amalgamation of individuals from mental health, academic and accommodation services meant all involved were not only aware of the individual’s needs and overarching support plan, but also of the practicalities involved in accessing support within different situations. “I met with my care coordinator, my course leader and someone from Resi’ Life, and we kind of all, spoke about the plan and that sort of thing, so that was really helpful” (Patricia, Y1).

*Informal support.* Those participants who were able to build understanding, accepting and trusting peer relationships tended to feel better supported overall, mitigating some of the systemic challenges above: “There was a while in first term when things were really really bad…I was really lucky that I’d met some amazing friends who…kind of knew when I wasn’t OK” (Angela, Y2).

Such friendships were crucial for participants, as they were able to be open about their difficulties rather than dealing with them in isolation, which could lead to deteriorating mental health. Furthermore, their friends actively helped them navigate university life, avoiding sensory sensitivities, reducing social anxiety, and even scoping out quiet spaces for those who needed them – “[my friend] finds places she knows I will hide if I get overwhelmed, so I don’t panic in the moment and know where I can go” (Angela, Y2).

### Recommendations

Participants were asked explicitly how they felt university mental health support could be improved. Answers spanned both the formal and informal aspects of university life.

Simplifying complex and difficult-to-navigate student support systems was a common recommendation: “If there was like a really quick, easy way you could reach out to services, even just alert these services like “I need help”” (Teresa, Y1).

One individual suggested an “initial consultation” (Emily, Y1) to assess needs, and in the same vein, another recommended a questionnaire to outline support needs so that staff knew someone “might need things explained to them a different way” (Louise, Y2). Others suggested that, for those who struggled to build friendships easily, more structured peer support could be beneficial: “I think we should have some sort of mentoring system, where you can get paired up with, all the students that have the same disabilities as you or like similar ones” (Janet, Y3).

Consistently, participants advocated autism awareness training to achieve the course adjustments and tailored mental health support which would be beneficial for them. However, awareness alone is not enough – it needs to be translated into action. Suggestions from students included a “campaign about how mental health issues can present differently” (Louise, Y2) in autistic people, “hiring autistic staff who know what it’s like” (Edward, Y1), asking autistic people to train university staff “about their experiences … what would have made it better for them” (Janet, Y3) and meeting the need for specialists in *both* mental health and autism within universities and the community more broadly: “The community mental health teams are, mental health and not so much autism … I think that would be good if there was some kind of specialist in that” (Patricia, Y1).

Finally, participants noted ways in which university events could be made more accessible, reducing the stress these currently place on their mental health. Large university events such as the fresher’s fair and careers fairs, and even particularly large lectures, were inaccessible to many, described as “very very very chaotic” and thus “really difficult” (Emily, Y1). Even the purported ‘quiet hour’ presented challenges because “everyone’s setting up their stalls … you just feel like you’re getting in the way if you go early” (Janet, Y3). Participants recommended more strictly limiting those allowed to enter these quiet hours, while structuring the day and supporting people more appropriately, such as providing “more information in advance, a better map…a bit of general information for people to be aware of people that are anxious, let alone autistic” (Emily, Y1). Recommendations from students for help with lectures similarly focused on better preparation, such as being allowed into a room early to “prepare themselves mentally” (Edward, Y1) and making sure that things like notes are available as soon as possible after the session “to help with my anxiety” (Emily, Y1), as this would make the lecture experience more predictable.

Overall, it was clear from talking to autistic students that their mental health varies across their time at university, but that there are common themes in what helps, and what hinders, their wellbeing. The significance of any challenges for mental health, particularly in the early stages of university and during transitions between terms, rested largely upon the nature of individual’s relationships with peers and staff, as well as access to appropriate support. Where good relationships were present, other elements came together to create an overall positive experience, but these could be undermined by a lack of autism knowledge among staff and a fear of being seen as a stereotype of an autistic person rather than as an individual.

## Discussion

This study presents a qualitative examination of autistic student’s experiences of mental health and mental health support at a single university. While the transition to higher education represents a challenge for autistic students and *can* intensify any existing mental health difficulties (Jackson et al., 2018), many participants in the present study reported success in managing the shift to a more independent way of living and studying.

Relationships appeared key to this success – tying together the positive and challenging elements of university for participants, for better and for worse. There was disparity between different participants’ mental health and confidence in seeking support based upon the nature of these relationships. For example, individuals who reported negative interpersonal experiences with staff relating to (lack of) autism awareness often spoke of a reluctance to seek autism-specific support at university for fear of ‘being treated differently’. Indeed, stigmatising attitudes towards autism – irrespective of self-reported awareness and openness - continue to prevail (Stronach et al., 2019), and some participants in the present study reported encountering such insensitivities among university staff.

However, others felt well-supported by autism-knowledgeable tutors and staff members. Accordingly, these students had been confident in not only accessing mental health support, but also asking for reasonable adjustments to make their overall university experience better. Encountering positive attitudes from staff, and acceptance from peers, was named as a crucial factor in supporting good mental health among our sample, as has been highlighted in other work on the positive impact of reduced autism stigma and increased community identity (Cage et al., 2018).

Many participants reported overall satisfaction with their peer relationships and were positive regarding those relationships’ impact on their mental health. This finding fits with quantitative evidence suggesting that the presence and number of friendships has ‘unique independent positive effects’ on mental health outcomes including anxiety, depression and self-esteem – even when moderated by self-reported loneliness and autistic symptomology (Mazurek, 2014; Reed et al., 2016). These findings stand contrary to theories which broadly conceptualise autistic people as lacking social motivation (Chevallier et al., 2012). Indeed, even individuals in the present study who reported feelings of isolation expressed their *desire* to make friends, but faced challenges overcoming barriers related to the intensity of social environments such as halls and fresher’s week. These difficulties may stem from social anxiety underpinned by intolerance of uncertainty, alexithymia and sensory hypersensitivities (Pickard et al., 2020).

Contrastingly, participants often formed positive relationships within societies – if able to access them. Recent qualitative research suggests that autistic women – the largest group in our study – place heightened value on friends that ‘let you be yourself’ and less on forming relationships to ‘look cool’ when compared to neurotypical girls of the same age (Cage et al., 2016; Sedgewick et al., 2019). Participants in the present study noted improvements in mental health due to no longer having to ‘mask’ their autistic traits and feeling accepted by peers. Masking (consciously and unconsciously reducing the visibility of autistic traits (Hull et al., 2019) is increasingly being identified as a specific and unique risk factor for poor mental health, self-harm, and suicidality among autistic people (Cassidy et al., 2018). That our participants felt their relationships were secure and accepting enough for them to drop these masks is highly encouraging and highlights the importance of relationships in higher education for creating positive experiences for our students. Indeed, support and loneliness act as protective and risk factors for depression and suicidal ideation respectively in autistic people (Uljarević et al., 2018).

University is a period where students gain freedom and control of their lives, but also take on the responsibility for managing accompanying increased self-sufficiency. Challenges with independence in our sample involved the management of mental health while adapting to this new way of life. Some suggest that such challenges with adaptation may be heavily influenced by unfamiliarity with a setting, and associated inconsistency in routine (Volkmar et al., 2017). This heightened unpredictability appears consistent with theories that situate Intolerance of Uncertainty as a key mechanism underlying anxiety – especially for autistic people (Boulter et al., 2014) – and it is logical therefore that adaptation demands affected mental health in our sample.

Participant’s ‘spikes’ in mental health issues around times of transition – along with the practical challenges seen in other research (Lambe et al., 2019) – also make sense in this light. Transitions are challenging for autistic people (Cheak-Zamora et al., 2015), and for students who experienced such ‘spikes’, the return to university represented a return to the freedom to act as they wished, which for some meant worsened self-harm and EDs. This pattern is also present in other populations – non-autistic individuals who engage in self-harm are likely to take more extreme measures following a period of abstinence (Davis & Lewis, 2019), and those with EDs may relapse following forced weight gain (Carter et al., 2012).

For our participants, however, the increased independence and control over their lives often supported positive mental health, as they were able to regulate their activities and interactions with others in ways that suited them. Giving autistic people autonomy has been shown to have positive effects on wellbeing (Milton & Sims, 2016), and the same effects were present for university students. Furthermore, the opportunity to engage intensely with a preferred subject has been shown to be appreciated in other autistic student samples (Ward & Webster, 2018), and the positive impacts of exploring special interests – which may be the focus of a degree – are beginning to be recognized in research (Grove et al., 2018; Wood, 2019).

The third broad theme - support - was split into formal (the organisation and appropriateness of mental health support) and informal support (personal relationships). While the positive influence of friendships on mental health has been discussed above, formal, systemic factors are a crucial area where institution-level action can be taken to significantly improve mental health support for autistic students. The aspects drawn out in this study – transitions between mental health teams, difficulty accessing support, lack of autism awareness among mental health staff, and lack of autism-tailored approaches – directly map onto the issues from recent work on mental health among autistic young people (Camm-Crosbie et al., 2019; Crane et al., 2019). Accessing support was one of the key difficulties for participants, as university systems were perceived as labyrinthine and disparate, requiring high levels of effort to navigate. However, with a supportive Personal Tutor, these challenges could be overcome, as seen in other work with autistic students (Cage & Howes, 2020).

Autistic students, however, should not be reliant on the ‘luck of the draw’ regarding the knowledge and attitude of their Personal Tutor. To this end, one of the main recommendations for improving support indicated the need for autism training among staff, alongside making support and university infrastructure more accessible. Several participants experienced damage to their mental health due to a lack of autism awareness in positions of responsibility – an issue reportedly widespread within organisations (Dillenburger et al., 2013). Changing these attitudes and rectifying the lack of autism acceptance these incidents represent is a crucial step in improving the experiences of current autistic students, and in making higher education a welcoming space for future applicants. While to date there are few published studies on the effectiveness of autism awareness training, one did find that a six-hour course co-delivered by an autistic person led to increases in autism knowledge, acceptance and ability to provide effective support (Hamilton et al., 2016). A recent study has shown that autism acceptance training can improve explicit – though not implicit – biases around autism, with potential improvements in how those who receive training respond to autistic people at work and in their lives generally (Jones et al., 2021). This shows the importance of academic and pastoral staff who work with autistic students completing autism awareness training, to hopefully stop the stigmatizing attitudes some of our participants had encountered.

Not only is it vital that mental health support services begin to offer autism specific support and receive autism training, but universities more generally should factor the needs of autistic people - and those with other disabilities - into their provision of accessible events and structures. This idea can be linked to the theory of universal design (Mcguire et al., 2006), which states that designing a service or space for those who are likely to have the most difficulties also generates improvements for all other users, rather than being an additional burden (Burgstahler & Russo-Gleicher, 2015). Creating accessible and comfortable spaces for autistic students has the potential to significantly improve their university experience, and therefore their mental health, with minimal institutional effort – many students simply wanted agreed accommodations to be executed properly, rather than asking for additional changes.

The initial transition to university was often mentioned as a key time of potential crisis. Importantly however, participants who took more time to prepare for university generally had more positive transition experiences. Support programmes for these transitions are growing in the UK, and autistic secondary school students gave positive feedback on the preparation a ‘transition to university’ programme around social, academic and independence-related challenges of university (Lei et al., 2020). While such evidence is encouraging, longitudinal work is needed to examine the impacts on wider university experiences. Furthermore, there is some evidence that peer mentoring and support group programmes, an idea discussed by our participants, can also improve the social integration of autistic students, with their concomitant benefits (Ashbaugh et al., 2017; Hillier et al., 2018; Siew et al., 2017).

## Limitations

As with any research, there were limitations to this study. Firstly, the small sample size, while not unusual for in-depth qualitative work with autistic people, means that our findings should not be over-generalised (Braun & Clarke, 2019). However, this serves to highlight avenues for future research – focusing on specific cohorts of autistic students who face challenges unique to their subject areas and educational career stage, such as PhD students. Secondly, the sample is highly homogenous in that most participants were white, used verbal communication, and attended the same highly academic research-focussed university in the United Kingdom. It is highly likely that autistic people of colour, for example, face additional barriers to entering higher education and accessing support while in higher education, and are likely therefore to have additional reflections to share on the topic. Similarly, those who use non-verbal communication will have different challenges socializing and forming the relationships identified as so important in this study. Despite these limitations, more autistic young people are attending higher education than previously, and the experiences of this academically capable group with minimal linguistic difficulties are represented in research. Thirdly, this work is by necessity cross-sectional. The findings in this study are therefore not generalizable, but instead present a set of potential avenues for other projects to take further and explore more deeply. Future research should seek to involve larger numbers of students in longitudinal work covering the whole of their degree to generate more in-depth knowledge of how mental health changes over time for this potentially vulnerable group.

## Conclusions

The current study is one of the first to examine the positive aspects of university for autistic students and their mental health, rather than focusing exclusively on difficulties. This approach emphasizes that autistic students enjoy positive experiences as well as challenges at university. Evaluating these accounts from *current* university students gives a more holistic view of autistic student experiences in terms of mental health. This gives new insights into the ways in which autistic students build relationships to create successful independent lives for themselves, something often missing from research narratives. The study is also unique in the level of autistic student involvement throughout the research process, something which both academics and students have found valuable. We would recommend more researchers seek out genuine participatory methods in their work, as it has enriched our enquiries, our analysis, and our writing while giving students insight into research and the ‘other side’ of academia. Our findings highlight that institutions should work to remove barriers to accessing appropriate, autism-specific mental health support, while training staff to be confident and proactive in their approach to supporting autistic student’s needs. University structures should also be made more accessible – reducing the strain post-secondary study can place on autistic student’s mental health, thus combatting student attrition and poor employment outcomes.

## Supplemental Material

sj-pdf-1-dli-10.1177_23969415211010419 - Supplemental material for ‘I have more control over my life’: A qualitative exploration of challenges, opportunities, and support needs among autistic university students Click here for additional data file. Supplemental material, sj-pdf-1-dli-10.1177_23969415211010419 for ‘I have more control over my life’: A qualitative exploration of challenges, opportunities, and support needs among autistic university students by Matthew Scott and Felicity Sedgewick in Autism & Developmental Language Impairments
