# Corrigendum to “‘I have more control over my life’: A qualitative exploration of challenges, opportunities, and support needs among autistic university students”

**DOI:** 10.1177/23969415211028689

**Published:** 2021-06-24

Scott, M., & Sedgewick, F. (2021). ‘I have more control over my life’: A qualitative exploration of challenges, opportunities, and support needs among autistic university students. *Autism & Developmental Language Impairments*, 6, 1–14. https://doi.org/10.1177/23969415211010419

In this paper, funding information was inadvertently missed. The correct statement is as given below:

