# Supplementary material for: ‘I have more control over my life’: A qualitative exploration of challenges, opportunities, and support needs among autistic university students
Source: Autism Dev Lang Impair. 2021 May 17;6:23969415211010419. doi: 10.1177/23969415211010419 (PMC9685136; doi:10.1177/23969415211010419)
Supplement: sj-pdf-1-dli-10.1177_23969415211010419 - Supplemental material for ‘I have more control over my life’: A qualitative exploration of challenges, opportunities, and support needs among autistic university students [file sj-pdf-1-dli-10.1177_23969415211010419.pdf]

EBI Mental Health Interview Schedule

- First, can you tell me a bit about your mental health?
  - Have you had any issues with anxiety or depression for example?
  - What about any other difficulties?
  - When do you first remember feeling that way/having those thoughts?

- Did anything change about your mental health when you came to university?
  - Did your mental health get better or worse when you came to university?

- What aspects of university did you find had negative impacts on your mental health?
  - For example, did you find the work expectations difficult?
  - What about social expectations?
  - How was it moving away from home and being more independent?

- What aspects of university had positive impacts on your mental health?
  - For example, did you prefer focussing on one subject you liked compared to several subjects at school?
  - What about social opportunities?
  - And how was it being more independent?

- Have these things changed over your time at the university?
  - Are certain periods more difficult than others for you?

- Have you asked anyone for help with your mental health while you've been at university?
  - Who did you ask?
  - When?
  - Why did you decide to ask for help?
  - If no – why didn't you ask for help?

- How useful was the help you received?
  - Did you find the guidance easy to understand?
  - Did you feel the approach worked for you?
  - Was the person you talked to aware that you are autistic?
  - Did they do anything specific to accommodate your needs?

- What would you change to improve the mental health support offered by the university?

- Is there anything else you want to tell me about your mental health and your time at Bristol?
